# Health status and health needs of older refugees from Syria in Lebanon

**DOI:** 10.1186/s13031-014-0029-y

**Published:** 2015-04-09

**Authors:** Jonathan Strong, Christopher Varady, Najla Chahda, Shannon Doocy, Gilbert Burnham

**Affiliations:** Center for Refugee and Disaster Response, the Johns Hopkins Bloomberg School of Public Health, 615 N Wolfe Street E8132, Baltimore, MD 21205 USA; Caritas Lebanon Migrant Center (CLMC), PO Box 55455, Sin El Fil, Lebanon

**Keywords:** Refugees, Syrians, Older populations, Disabilities, Non-communicable diseases, Lebanon

## Abstract

**Background:**

The flight of Syrian and Palestinian families into Lebanon from Syria included a number of older refugees. This study sought to characterize the physical and emotional conditions, dietary habits, coping practices, and living conditions of this elderly population arriving in Lebanon between March 2011 and March 2013.

**Methods:**

A systematic selection of 210 older refugees from Syria was drawn from a listing of 1800 refugees over age 60 receiving assistance from the Caritas Lebanon Migrant Center (CLMC) or the Palestinian Women’s Humanitarian Organization (PALWHO). CLMC and PALWHO social workers collected qualitative and quantitative information during 2013.

**Results:**

Two-thirds of older refugees described their health status as poor or very poor. Most reported at least one non-communicable disease, with 60% having hypertension, 47% reporting diabetes, and 30% indicating some form of heart disease. Difficulties in affording medicines were reported by 87%. Physicial limitations were common: 47% reported difficulty walking and 24% reported vision loss. About 10% were physically unable to leave their homes and 4% were bedridden. Most required medical aids such as walking canes and eyeglasses. Diet was inadequate with older refugees reporting regularly reducing portion sizes, skipping meals, and limiting intake of fruits, vegetables, and meats. Often this was done to provide more food to younger family members. Some 61% of refugees reported feeling anxious, and significant proportions of older persons reported feelings of depression, loneliness, and believing they were a burden to their families. 74% of older refugees indicated varying degrees of dependency on humanitarian assistance.

**Conclusion:**

The study concluded older refugees from Syria are a highly vulnerable population needing health surveillance and targeted assistance. Programs assisting vulnerable populations may concentrate services on women and children leaving the elderly overlooked.

## Background

Older persons face a variety of age-specific disadvantages during times of conflict and displacement. Many hesitate to leave their communities even though staying places them at increased risk of injury and death. Among those who do leave, some fail to safely reach their destination. Impairments in mobility, vision, hearing, memory, and cognition lead to increased dependence on others, yet social networks are often disrupted, support from family may diminish, and esteem, once held in home communities, may decline. Pensions may stop, and their investment in housing, businesses, or other property may be lost. Medical services such as primary healthcare, medications, and medical devices such as hearing aids needed for aging-associated diseases and impairments may not be available or deemed not to be a priority. Although services for vulnerable populations affected by conflict and displacement are emphasized, this is usually translated as extra assistance to women and children [1].

From 2005 to 2010, worldwide the population of those aged 60 or older grew twice as fast as all other age groups due to decreasing fertility rates and increased longevity [2]. These demographic changes result in relatively fewer caretakers available to care for expanding older populations. Even in non-conflict conditions, economic circumstances for older populations are tenuous particularly in low and middle income countries (LMICs), where nearly two-thirds of all older adults live [3]. Among those older adults residing in LMICs, 80% have no regular income [4].

An estimated 26 million older persons are affected by natural disasters every year [5]. Older persons also now constitute 8.5% of UNHCR’s Population of Concern, a number much higher than generally appreciated [6]. LMICs already suffer disproportionately from disasters with 97% of those killed in natural disasters living in those countries [6-9]. The Pan American Health Organization notes that in disasters, older persons with a progressive loss of function can have difficulty adapting to the challenges and coping with disruptions [10].

Several reports have documented the circumstances of older refugees in a variety of conflict and displacement settings. Among Rwandan refugees in Tanzania, 19.3% of men over age 60 and 13.1% of women over age 60 were found to have a Body Mass Index (BMI) of 18.5 or less, rates higher than among younger adults [11]. This study found that loss of social networks, loss of esteem, restricted mobility, lack of access to food rations, and lack of access to basic supplies contributed to nutritional vulnerability [12]. Even with nutritional access, older persons may have difficulties opening packaging, preparing food or feeding themselves [13]. The poor nutritional status of the older refugees contributes to poor health and limits their ability for self-care. Those living in social isolation are particularly vulnerable [14].

This paper reports recent findings among older refugees from Syria now residing in Lebanon. The over-60 population of Syria is estimated at 5.8% [15]. This population is perhaps the least able to cope among the 3.9 million refugees now outside Syria [16]. At the time of the study, UNCHR estimated that there were nearly 400 thousand Syrian refugees in Lebanon [17]. Agencies in Lebanon have reported increasing numbers of older Syrian refugees needing assistance.

The Caritas Lebanon Migrant Center (CLMC) has been providing assistance to migrants in Lebanon for 20 years, assisting some 125,000 Syrian refugees since the start of the current crisis. This assistance includes the provision of food and non-food items such as blankets, tarpaulins, and cooking stoves; medical assistance through referrals and the Caritas Lebanon mobile clinic; psychosocial counseling for traumatized refugees; education assistance to children to enroll in Lebanese public schools; and legal assistance in matters of births, deaths, marriages, immigration, and repatriation to third countries. In pursuing these activities, CLMC has increasingly noted older refugees with poor health and limited support from families. To better understand the needs of the older refugees, the CLMC, with technical assistance from the Johns Hopkins Bloomberg School of Public Health (JHSPH), undertook a detailed evaluation of health status and living circumstances of 210 older refugees in Lebanon including both Syrians and Palestinians from Syria fleeing the current conflict. The study was carried out in January – March 2013 in populations that had been registered and receiving support from CLMC and the Palestinian Women’s Humanitarian Organization (PALWHO). Older Palestinian refugees were included to inform the policies and practices of PALWHO. It was not a primary aim of the study to draw comparisons between older Syrian refugees and older Palestinian refugees from Syria, but comparisons are drawn to provide additional information. The Institutional Review Board of the Université St. Joseph, Beirut, reviewed and approved the study. The JHSPH Health Institutional Review Board declared the analysis of data exempt.

## Methods

The study population consisted of refugees from Syria aged 60 and above registered with CLMC or PALWHO as of January 2013. A Syrian national or Palestinian refugee resident in Syria who entered Lebanon after March 2011 was considered a refugee for the purposes of this study. We restricted participation to older refugees fleeing from Syria, as there are many migrant workers with older family members from Syria who may have chosen to stay in Lebanon. Registration status with UNHCR or UNRWA was not an inclusion or exclusion criterion. For this study, we considered a household as a group eating together and functioning as a single economic unit.

Both qualitative and quantitative instruments were used to record data such as prior events, financial status, health status, and medical diagnoses. Medical diagnoses were identified by self-report and not verified by medical professionals. The Katz Index of Independence in Activities of Daily Living was used to assess functional status using six common functions [18].

Participants in this survey were drawn as a systematic sample from the CLMC and PALWHO databases of refugees from Syria that included approximately 1,100 older Syrian refugees registered with CLMC and 700 older Palestinian refugees from Syria registered with PALWHO as of January 2013. This ensured that the number of study participants selected at each CLMC field office (Baalbeck, Saida, Sin el Fil, Taalabaya, Tripoli, and Zahleh) and PALWHO field office (Bourj el-Barajneh, Mar Elias, and Shatila) was proportional to the number of older refugees registered at each field office. When interviewers at a particular site could not locate a selected refugee, the next nearest refugee to that residence site meeting study inclusion criteria was selected. The study sought to include 220 older refugees from Syria including 175 older Syrian refugees and 45 elderly Palestinian refugees. Sample size was determined by seeking the greatest number of older refugees possible with the limited finances and staff dedicated to the study by CLMC and PALWHO. These sample sizes allowed for the measurement of population characteristics within a margin of error of ±7.6% among older Syrian refugees and ±15% among older Palestinian refugees. The calculation of margin of error assumes the most conservative prevalence rate of 50%, a survey response rate of 95%, a study design effect of 1.0, and a 95% confidence interval.

The survey questionnaire was field tested by speaking to approximately 15 elderly Syrian refugees residing in areas surrounding the CLMC field office in Taalabaya. Interviews were conducted in Arabic by CLMC and PALWHO social workers in 2013 after five days of training. Written informed consent was performed prior to administration of the survey questionnaire. Data was recorded on paper forms in the field. Data entry and data analysis was performed in Beirut.

## Findings

**Figure 1:**
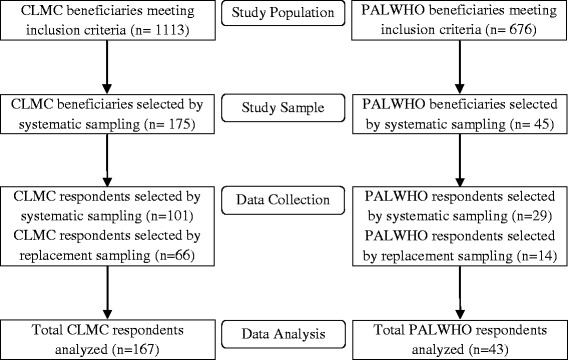
**Sampling methodology and response rate.**

**Figure 2:**
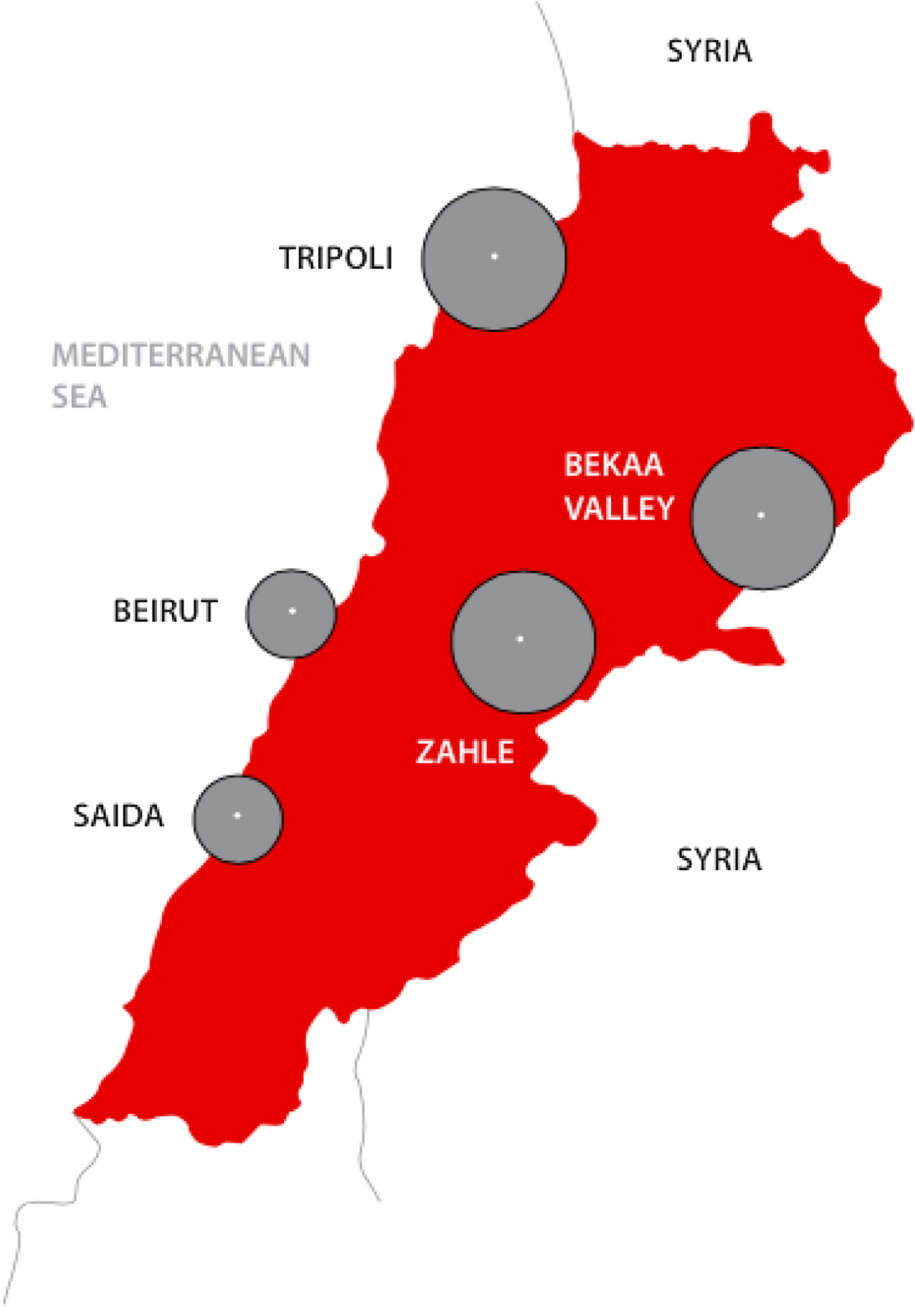
**Geographic distribution of survey respondents.** Size of circle is proportional to the number of elderly refugees sampled.

Sampling methodology and response rate is displayed in Figure 1. 167 older Syrian refugees and 43 older Palestinian refugees were interviewed, representing response rates of 95.4% and 95.5%, respectively. Because of continuing movement of refugees within Lebanon, 39.5% of older Syrian refugees and 31.1% of older Palestinian refugees participating in this study were selected by replacement sampling. The geographic distribution of respondents was roughly in proportion to the geographic distribution of older refugees in the CLMC and PALWHO databases, with the exception of some oversampling of older Syrian refugees in the Mt. Lebanon region due to many older refugees expressing great interest in the study in this region. Table 1 displays the geographic distribution of Syrian respondents and, for reference, the distribution of older UNHCR-registered Syrian refugees at the time of the survey. The 43 Palestinian respondents resided in Beirut, with the majority (30) in the Burj el Barajineh camp. Figure 2 depicts a map of locations in which elderly refugees were sampled.

**Table 1:** **Geographic distribution of older Syrian survey respondents and estimated number of older Syrian refugees registered or awaiting registration with UNHCR as of March 29 2013**

**Region**	**Syrian survey respondents (n)**	UNHCR estimated refugees 60 years or older* (n)
Bekaa	70% (117)	34% (3837)
North	12% (20)	34% (3777)
Beirut/Mt. Lebanon	11% (18)	17% (1942)
South	7% (12)	14% (1601)
Total	100% (167)	100% (11157)

*Estimates of older refugees registered or awaiting registration with UNHCR assumes the age distribution of Syrian refugees does not vary by geographic region in Lebanon.

The average age of Syrian refugees was 68 years old. The median age was 66 years and the age range was 60–96 years with 22% aged 75 and above. On average, Palestinian refugees were 4.6 years older than Syrian refugees. Males constituted 50.9% of Syrian refugees but only 17.1% of the Syrian Palestinian refugees sampled. The PALWHO database used to identify elderly Palestinians serves as the basis for various programs focused on providing assistance to women, affecting this proportion. Half of elderly Syrians were illiterate compared to 21% of Syrian Palestinian refugees.

### Place of origin in Syria

Data about place of origin in Syria is incomplete, as many feared giving this information. Among Syrians who responded (n = 66), 32% came from Homs, 26% from Damascus, and 20% from Aleppo. For Palestinians from Syria (n = 43), most came from Damascus (45%), Daraa (19%), or Idlib (17%), with 36% living in a refugee camp in Syria. At the time of the survey over half of the Syrians had been in Lebanon for more than seven months. The Palestinians were on average more recent arrivals.

### Leaving Syria

A majority of older refugees reported that their neighborhoods in Syria had been under heavy bombardment or were surrounded by fighting. Many said their homes were badly damaged or destroyed. Some families reported being trapped for several weeks or months, waiting for a break in the fighting to escape to Lebanon. Older Syrians frequently mentioned that family members, usually sons, were killed in the fighting, imprisoned, or disappeared for months or even longer. Those fleeing Syria at later dates had more accounts of violence than those fleeing earlier [p <0.001]. However, some elderly left for reasons indirectly related to the violence such as the unavailability of necessary medical supplies for problems such as heart disease or chronic pain. These older refugees stated that the hospitals and clinics that were their usual source of care had been closed or destroyed, and the cost of medicine had increased dramatically as supplies in Syria ran low. Others came to Lebanon in search of food or water, necessities which had become scarce in Syria.

Palestinians were more likely to report problems crossing into Lebanon. Many stated they had waited several days for a visa, and others admitted not knowing the procedures to cross the border. Some elderly Palestinian refugees took loans or sold assets such as sheep or jewelry to pay the formal and informal costs of the border crossing.

Only about half of the older Syrian refugees were registered with UNHCR, but an additional 15% had applications pending at the time of interview. While some were confused about the registration process, many had not registered due to fears of being identified and having their personal details recorded. There was no association between UNHCR registration and self-reported financial status [*p* = 0.142], self-reported health status [*p* = 0.204], change in health status since arriving in Lebanon [*p* = 0.579], ability to see a doctor [*p* = 0.646], ability to access needed medications [p = 0.192], number of non-food items needed [*p* = 0.074], number of days in the past week meal portions were reduced due to lack of food [*p* = 0.086], number of days in the past week a meal was skipped due to lack of food [*p* = 0.125], and number of days in the past week in which no food was eaten due to lack of food [*p* = 0.992].

### Household living conditions

Older Syrian refugees lived in houses (39%), in tents (26%), in apartments (23%), and public buildings, unfinished structures, or other dwellings sites (11%). Most Palestinians found houses or apartments, but some lived in unfinished buildings or public structures, generally located in existing Palestinian refugee camps. The average size of older Syrian refugee households was seven persons with 12% of households having more than 12 or more people. Palestinian households were significantly larger: the average household size was 10.5 people, and 52% of households had 12 or more people. The majority of older Syrian refugees were married (72%) or widowed (24%), with 3% reporting their spouse had stayed in Syria. However, among Palestinian refugees from Syria, 47% were married, 37% were widowed, and 16% were divorced, separated, or never married.

Approximately 40% of older refugees were themselves providing physical care for someone in their household with 37% providing care for their spouse and 32% providing care for a child aged 5 to 15 years old. Less commonly, older Syrian refugees cared for other older adults (18%), other non-elderly adults (8%), or children under age five (5%). Care-giving practices were less common among Palestinian refugees from Syria, though there were may have been problems with how the question was asked.

Older refugees were asked about the reasons why family and friends remained behind in Syria. Common responses included the lack of finances necessary to leave, the inability to leave safely due to conflict, the necessity of protecting a house or other assets, and limitation due to physical disabilities.

### Financial conditions

Older refugees reported financial difficulties living in Lebanon, with 74% saying they depended on receiving financial help or humanitarian aid to provide basic necessities such as food, water, shelter, or medicine. A quarter said they could usually afford basic necessities but sometimes had to borrow, rely upon humanitarian aid, or go without. Only 2% of elderly refugee households were able to consistently afford necessities using their own finances. About 22% of older refugees relied on their family as their principal means of support. Although financial circumstances varied widely among households, the older Palestinian refugees seemed to have greater financial difficulties.

### Self-reported health status

The majority of older refugees reported their health as poor (54%) or very poor (12%). Self-reported health status was worse among those with less education and the very old. A majority indicated that their health has gotten worse since coming to Lebanon (66%). There were no significant differences between Syrians and Palestinians with regards to self-reported health status or change in health since coming to Lebanon.

### Non-communicable diseases (NCDs)

Older refugees reported a high burden of chronic illnesses and disabilities as depicted in Table 2. Hypertension was most common (60%), followed by diabetes mellitus (47%), and heart disease (30%). The burden from these diseases was significantly higher in older Palestinians compared to older Syrians, even when controlling for the effects of sex and age. [hypertension *p* < 0.001; diabetes *p* < 0.001; heart disease *p*=0.042] Other common conditions reported were high cholesterol; musculoskeletal conditions as arthritis, injury or back pain; eye disease (not eyeglasses); and chronic pain. Palestinian refugees from Syria reported an average of 4.0 NCDs, and the Syrian refugees an average of 2.5 NCDs, a difference that was significant when controlling for differences in sex and age [*p* <0.001].

**Table 2:** **Non-communicable diseases reported by older refugees from Syria**

**Non-communicable disease**	**Syrians (n = 167)**		**Palestinians (n = 43)**		**p-value** ^*^
	**Point estimate**	**95% CI**	**Point estimate**	**95% CI**	
Hypertension	53%	46–61%	86%	72–94%	**<0.001**
Diabetes mellitus	38%	55–69%	81%	66–91%	**<0.001**
Heart disease^a^	28%	22–36%	40%	26–55%	0.148
High cholesterol	22%	16–29%	42%	28–58%	**0.007**
Arthritis, injury, or back pain	31%	24–38%	7%	2–20%	**0.002**
Eye disease (not eyeglasses)^b^	16%	11–22%	28%	16–44%	0.061
Chronic pain	15%	10–21%	9%	3–23%	0.337
Lung disease^c^	11%	7–17%	44%	30–60%	**<0.001**
Digestive tract disease^d^	9%	5–14%	23%	13–39%	**0.010**
Neurologic disease^e^	5%	2–9%	12%	5–26%	0.097
Renal disease^f^	6%	3–11%	7%	2–20%	0.810

^*^Chi-squared test, statistical significance if p-value of <0.05.

^a^Heart disease includes coronary artery disease, valvular heart disease, heart failure, and arrhythmia.

^b^Eye disease includes retinal cataracts, glaucoma, retinal disease, and eye injuries but not eyeglasses.

^c^Lung disease includes asthma, COPD, chronic cough, and interstitial lung disease.

^d^Digestive tract disease includes diseases of the esophagus, stomach, bowel, rectum, liver, gallbladder and pancreas.

^e^Neurologic disease includes stroke, epilepsy, headache, and vertigo.

^f^Renal disease includes renal failure, nephrolithiasis, or other disease of the urinary tract.

### Access to care

Financial difficulties were given as the primary reason for not seeking care by 79% of older refugees. Other barriers included lack of knowledge about where to seek care (12%) and the physical inability to travel to a heath facility (4%). Only 1.5% stated they had no difficulties in obtaining care when needed. When asked about access to medicines, 87% reported they had difficulty affording the cost of medication, 7% reported they did not know where to buy medication, and 3% said they were physically unable to go to the pharmacy. Only 1.5% reported that they had good access to care and 3% reported sufficient money to buy medicines.

Many older refugees were still taking medicine they brought from Syria and did not believe they could afford higher priced medicines in Lebanon. Some arranged for medicines to be brought to them from Syria, however this was often unreliable. Others had stopped taking the medicines when supplies from Syria finished.

### Physical limitations and disabilities

Physical limitations and disabilities are reported in Table 3. Difficulty walking was the most common physical limitation reported (44%), followed by vision loss (24%) and hearing loss (18%). Many older persons reported more than one physical limitation or disability. Approximately 10% of older refugees were physically unable to leave their house or shelter and 4% were bedridden. Physical limitations were more common among Palestinians when controlling for age and sex [difficulty walking *p*=0.002; vision loss *p* <0.001; hearing loss *p* <0.001]. Access to items such as eyeglasses, hearing aids, hygiene supplies and mobility devices in Lebanon was perceived as inaccessible because of price. Perceived needs for these items were seen as much greater among Palestinian refugees from Syria, as measured by the number of needed items [*p* <0.001].

**Table 3:** **Physical limitations reported by older refugees from Syria**

**Physical limitation**	**Syrians (n = 167)**		**Palestinians (n = 43)**		**p-value** ^*^
	**Point estimate**	**95% CI**	**Point estimate**	**95% CI**	
Difficulty walking	39%	32–47%	65%	49–78%	**0.002**
Impaired vision	13%	8–19%	70%	54–82%	**<0.001**
Impaired hearing	9%	6–15%	49%	34–64%	**<0.001**
Physically unable to leave the home	8%	5–13%	16%	8–31%	0.894

^*^Chi-squared test, statistical significance if p-value of <0.05.

### Functional status

Many older refugees were dependent on others for dressing (26%), bathing (26%), using the toilet (22%), transferring positions (21%), maintaining continence (20%), and feeding (12%). However, 64% reported they were fully independent in performing all six activities. There were 10% who were moderately impaired (requiring help with 2–3 activities) and 18% were severely impaired (requiring help with four or more activities). Poorer functional status was associated with advanced age, the presence of dementia, poor vision, difficulty walking, poor reported health status, and larger household size. [age *p*=0.002; dementia *p*=0.001; poor vision *p*=0.018; difficulty walking *p*=0.029; health status *p*=0.009; household size *p*=0.003] There was a trend towards lower functional status among females and among Palestinians, but this was not statistically significant.

Nearly all older refugees – 96% of Syrians and 100% of Palestinians – reported they had a family member who would take care of them if they were sick or help them if they had an emergency. Most Syrian refugees also had a friend who could take care for them or help in an emergency. Only half of the Palestinians refugees felt they would have this help. Daughters-in-law were reported as the most common caretaker. Some elderly cited other elderly family members who would provide assistance when needed. In the case of multiple wives, the youngest wife would most often be the caretaker to the husband. In several cases this led to the neglect of the needs of older wives.

Did older refugees feel as if they were a burden to their families? Perceptions varied. Older Syrian refugees who were able to assist in child care or household chores usually did not feel as if they were a burden to their families. However, very old Syrian refugees and those who were who were disabled or unable to perform activities of daily living without the help of family members usually did perceive themselves a burden to their families. Some expressed guilt that they were unable to help with household chores or provide financially for the family. In contrast, almost no older Palestinian refugees from Syria expressed feelings of being burdensome to their family. One older Palestinian explained that in their culture it is expected that younger family members provide for the older family members.

### Diet

**Figure 3:**
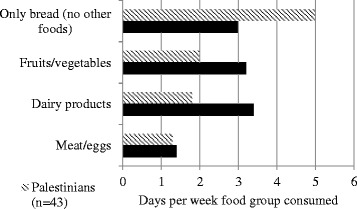
**Reported days in the previous week that various foods were consumed.**

Older refugees were asked how many days out of the past week they had consumed certain foods including meat or eggs, dairy products, and fruits/vegetables (Figure 3).

The number of days older refugees reporting eating bread only and nothing else corresponded to their reported financial status. [*p*=0.036] Similarly, older refugees living in large households with many others consumed only bread more frequently and other food groups (such as meat, dairy, fruits, and vegetables) less frequently than did older refugees living in smaller households [only bread *p* <0.001; meat *p*=0.013; dairy *p* < 0.001; fruits and vegetables *p* < 0.001]. Older refugees living in tents ate fruits and vegetables more frequently and ate only bread less frequently than those living in houses. [fruits and vegetables *p* < 0.001; only bread *p* < 0.001] This may be because tent settlements are more likely to be in rural agricultural areas, whereas houses are more often in urban areas with easy access to bakeries. Accordingly, those living in apartments consumed only bread more frequently than those in houses. [*p*=0.002] Those living in public buildings had the least amount of diversity in their diets, eating meat, dairy, fruits and vegetables less frequently than those in houses. [meat *p*=0.015; dairy *p*=0.011; fruits and vegetables *p*=0.002].

**Figure 4:**
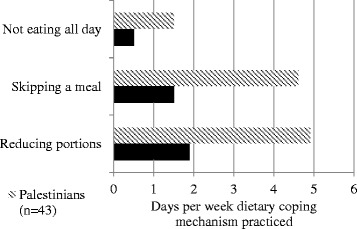
**Reported days in the previous week that dietary coping mechanisms were practiced.**

Older refugees were also asked how many days out of the past week they had reduced meal portion sizes, skipped a meal, or gone the entire day without eating due to lack of food (Figure 4). On average, older Palestinian refugees practiced these dietary coping strategies significantly more frequently than older Syrian refugees [reducing portion sizes *p* <0.001; skipping a meal *p* <0.001; not eating at all *p* <0.001]. Factors associated with reducing portion sizes or skipping meals included poor financial status, large household size, and type of residence [financial status *p*=0.009; household size *p* <0.001; type of residence *p* <0.001]. The majority (88.7%) of older refugees reported cost as their biggest problem related to food.

### Negative emotions

Older refugees were asked to identify negative emotions they may have felt in the past week (Table 4). Refugees were allowed to indicate having multiple negative feelings, and in fact for many older refugees this was common. Feelings of anxiety in older refugees were closely related to whether or not a friend or family member was available to help them in an emergency. [*p*=0.006] Feeling depressed was significantly more common among older refugees who were more educated and those more advanced in age [education *p* = 0.023, age *p*=0.017]. Feelings of loneliness among older refugees were associated with poor financial status, living in a tent or house, and lacking a friend who could provide care if the older refugee became sick [financial status *p*=0.035; living in a tent or house *p* <0.001; lacking a friend *p*=0.001]. Household size and marital status did not affect feelings of loneliness.

**Table 4:** **Negative emotions experienced by older refugees in the previous week**

**Negative emotion**	**Syrians (n = 167)**		**Palestinians (n = 43)**		**p-value** ^*^
	**Point estimate**	**95% CI**	**Point estimate**	**95% CI**	
Anxious	41%	34–49%	30%s	18–46%	0.184
Depressed	25%	19–32%	40%	26–55%	0.050
Feeling unsafe	24%	18–31%	23%	13–39%	0.924
Lonely	23%	17–30%	30%	18–46%	0.308
Scared	18%	13–25%	33%	20–48%	**0.036**
Irritable or angry	13%	9–19%	5%	1–18%	0.117

^*^Chi-squared test, statistical significance if p-value of <0.05.

During qualitative interviews, it became clear that many elderly refugees were discouraged about their current situation. Many related feelings of sadness, loss of appetite, decreased energy, difficulty sleeping, and loss of interest in things they used to enjoy. They felt powerless, yearning to return to Syria but knowing this was now impossible at the current time.

### Negative emotions and day-to-day functions

When asked about the impact of these negative emotions on their ability to function, 11% of older refugees reported that they function normally or almost normally and 57% said that negative feelings restricted their ability to perform some tasks, but that they were able to do at least half of what a healthy person of their age would be expected to do. There were 32% who said that their negative emotions cause serious restriction, impairing their ability to do at least half of what a healthy person of their age would be expected to do. Those with poor physical health and those with high levels of education were significantly more restricted by negative emotions [physical health *p* < 0.001; education *p*=0.015].

## Discussion

In refugee crises, very little attention is given to assessing the age-specific needs of older refugees. Even when data is collected, findings are not frequently translated into specific programming. This is also true for the current crisis with the influx of Syrian refugees. To fill these gaps of knowledge and practice, a systematic sample of older refugees drawn from the extensive databases of CLMC and PALWHO was interviewed in multiple locations in Lebanon. The majority of older refugees had been in Lebanon for 12 months at the time of interview. The situation of the older refugees was dire. Deaths and disappearances of younger males from the household often left many elderly refugees exposed and poorly supported by diminished families.

The older Syrian refugees had many physical limitations and chronic health problems. More than half of older refugees reported their heath was very bad and about two-thirds said their health has gotten worse since arriving in Lebanon. Despite having many physical limitations and chronic health problems – most commonly hypertension, diabetes, heart disease, difficulty walking, and impaired vision – nearly all older refugees reported they were unable to obtain adequate medical treatment. Cost was the primary barrier in seeking care – only 3% of older refugees reported having the financial resources to reliably afford basic necessities such as medicines. The prices of medicines were much more expensive in Lebanon compared to pre-conflict prices in Syria, causing many older refugees to rely on diminishing supplies of medications in hand or to forego taking medications to pay for other basic necessities. Going without medications for chronic conditions increases the risk of severe and difficult to treat complications. Indeed, one refugee reported that his elderly father had suffered a stroke after several months of foregoing medications for diabetes and hypertension and that he feared another stroke might occur as the cost of medications remained out of reach.

Food insecurity and poor dietary quality are also grave concerns for older refugees from Syria. Many older refugees, particularly older Palestinian refugees, frequently skip meals, go entire days without eating, or lack essential food groups such as fruits, vegetables, meats, and dairy. Compounding this problem is the high prevalence of chronic diseases such as hypertension and diabetes that require specialized diets to manage properly. Diets varied significantly by financial status, household size, and type of housing.

Reports of recurring negative emotions were common, particularly anxiety (39%), depression (28%), and loneliness (24%). For a significant proportion of older refugees, these negative emotions caused significant distress and interfered with their ability to perform day-to-day functions. During qualitative interviews, it became clear that many factors had contributed to these negative feelings including witnessing traumatic events, living in unsatisfactory living conditions, coping with poor physical health, lacking support of friends and family, and grieving for family members that were killed or disappeared.

The results of this study emphasize the importance of disaggregation of data to look at specific issues of the older populations. Much of the information needed to provide support that older refugees needed was available only with detailed questioning. Looking separately at issues of the Palestinian refugees from Syria showed evidence of greater vulnerabilities. While the selection process may have had some biases, particularly for women, nonetheless, the data points to the older Palestinians as being considerably worse off than their Syrian counterparts in almost every category, from financial conditions, to chronic diseases, to diet. These discrepancies between older Syrian and Palestinian refugees persisted when controlling for population differences in age, sex, and education level.

Although these data suggest many vulnerabilities and potential liabilities to households with older refugees, the older refugees provide many positive contributions to the household and to the larger refugee community. Older refugees in this study were active in providing childcare, particularly important where many household males were killed or absent and females assume responsibility for generating income. This older population also preserves cultural identity and values in unstable and volatile situations such as currently in Lebanon. Elders often can provide community arbitration using traditional problem solving approaches, especially in societies that value older persons. In these cultures the older age groups can speak for a community and command respect from the host community in a non-threatening manner.

There are a number of limitations to this study. The sample population, though we believe to be representative of older Syrian refugees, is drawn from those areas of Lebanon where CLMC provides services and the distribution does not match the locations where UNHCR has registered refugees. As the refugees enter Lebanon they continue to move. The specific refugees systematically selected from the CLMC records for interview were often not present at the field site when the team arrived. Replacements were selected from refugees matching the age criteria living in areas adjacent to the refugee original selected. Since age was the only selection criteria, this probably did not introduce bias. In one area, Mt Lebanon, there was a slight oversampling, thought this did not change the characteristics of the dataset. Finally, for the Palestinians a separate listing from PALWHO was used to identify older refugees. As this database was used for many women’s programs there was a likely bias toward the selection of women, and indeed, while the CLMC sample was 51% males, the PALWHO sample was only 17% males. The older mean age for Palestinians may have been related to this female preponderance.

The survey depended on recall, particularly for medical diagnoses, which may have been problematic for some. As there were a number of interviewers in different parts of Lebanon, there may have been so variations in interviews, especially the qualitative questions.

## Conclusions and recommendations

Despite their needs and vulnerabilities, older persons should not be seen as solely dependent or weak. Older persons bring specific assets and strengths to emergency settings, though they typically receive even less recognition for these than for their vulnerabilities. In situations of population displacement, they are often able to negotiate more effectively for space, housing, and tolerance from host communities. In fact, in the Middle East, older persons who speak on behalf of larger groups of refugees are more respected and more likely to be listened to by policymakers. Further, the elderly preserve and transmit traditions and customs that define a people’s cultural identity, even in times of social disintegration. This sense of identity can moderate extreme views during times of conflict, contributing to the peace-building process. Older persons often contribute significantly to household chores and childcare, particularly when a child’s parents are not present. The failure to recognize and leverage these contributions represents a missed opportunity not only to bolster the sense of self-worth among conflict-affected elderly, but also to better the families and communities where the elderly reside.
